# In Vivo Determination of Organellar pH Using a Universal Wavelength-Based Confocal Microscopy Approach

**DOI:** 10.1371/journal.pone.0033229

**Published:** 2012-03-21

**Authors:** Albert Pineda Rodó, Libuše Váchová, Zdena Palková

**Affiliations:** 1 Department of Genetics and Microbiology, Charles University, Prague, Czech Republic; 2 Institute of Microbiology, Academy of Sciences, Prague, Czech Republic; Université de Nice-CNRS, France

## Abstract

Many essential cellular processes are affected by transmembrane H^+^ gradients and intracellular pH (pHi). The research of such metabolic events calls for a non-invasive method to monitor pHi within individual subcellular compartments. We present a novel confocal microscopy approach for the determination of organellar pHi in living cells expressing pH-dependent ratiometric fluorescent proteins. Unlike conventional intensity-based fluorometry, our method relies on emission wavelength scans at single-organelle resolution to produce wavelength-based pH estimates both accurate and robust to low-signal artifacts. Analyses of Ato1p-pHluorin and Ato1p-mCherry yeast cells revealed previously unreported wavelength shifts in pHluorin emission which, together with ratiometric mCherry, allowed for high-precision quantification of actual physiological pH values and evidenced dynamic pHi changes throughout the different stages of yeast colony development. Additionally, comparative pH quantification of Ato1p-pHluorin and Met17p-pHluorin cells implied the existence of a significant pHi gradient between peripheral and internal cytoplasm of cells from colonies occurring in the ammonia-producing alkali developmental phase. Results represent a step forward in the study of pHi regulation and subcellular metabolic functions beyond the scope of this study.

## Introduction

The maintenance of intracellular pH (pHi) and homeostasis is fundamental in multiple cellular processes, as for instance transmembrane transport, establishment of electrochemical gradients, adaptive responses to environmental pH variations and other metabolic events taking place within particular organelles. The quantification of pH changes at the subcellular level is not easy, but if properly done it can be extremely useful in the physiological study of most living organisms. In contrast to more stable parameters, pHi is rapidly altered by almost any manipulation during sample measurement, including common methods for cell harvesting and fluorescent pH probe staining. For all these reasons, the monitoring procedure must be consistent to ensure the reproducibility and reliability of pH estimations. Some of the above requirements are addressed by incorporating genes encoding fluorescent proteins (FP), the fluorescence of which changes according to pH. Green fluorescent protein (GFP) from *Aequorea victoria* and most of its mutants exhibit some degree of fluorescence sensitivity to proton concentration and can be employed as noninvasive pH indicators [1]. Existing FPs can be classified as either ecliptic or ratiometric depending on their emission and excitation behavior: ecliptic FPs vary in intensity while ratiometric FPs shift their spectral wavelength. Metabolic and internal pH changes can be monitored in vivo by targeting FP fusion proteins to subcellular compartments and measuring its fluorescence changes through spectrofluorometry and confocal fluorescence microscopy (CFM) [2].

The accuracy of pH estimations strongly depends on the strength and nature of the pH response (intensity or wavelength) as well as dissociation constants (pKa) within the studied physiological pH range. The choice of pKa becomes critical at more extreme pH conditions such as the alkaline lumen of peroxisomes or acidic vacuoles, where the use of GFP-based indicators with a pKa close to neutrality for lack of a better option can lead to pH miscalculations. The ecliptic group, with a more diverse offer of pKa values, comprises the majority of pH-sensitive FPs including the widely used ecliptic pHluorin [3]. Nonetheless, when dealing with weak fluorescent signals it can be difficult to establish which intensity fluctuations are due to pH alone, since they are more susceptible to interference by photobleaching and autofluorescence. In such cases, pHi monitoring based solely on ecliptic intensity gradients or even more precise dual intensity ratios may not suffice for a fully quantitative measurement. Ratiometric fluorophores undergoing wavelength shifts in excitation or more preferably emission [4], [5] partly overcome these problems, but they are less common and therefore available quantum yields and pKa values are more limited.

In this study, we introduced a new confocal microscopy approach for the quantification of pHi in organelles within living cells (in situ) via direct measurement of emission wavelength changes from different fluorescent proteins. The utility of the approach was demonstrated by analyzing cells from *Saccharomyces cerevisiae* colonies passing through distinct developmental phases signaled by volatile ammonia (NH_3_) and supposedly associated with significant changes in extracellular and possibly intracellular pH [6], [7], [8]. The protocol relies on CFM imaging to discriminate fluorescent fractions within single cells, reduce photobleaching and amplify signal-to-noise ratios; ultimately enabling a broader selection of FPs, pKa values and allowing for higher precision measurements of organellar pH values near the extremes of the physiological scale. We proved that the new technique can be generally used for the empirical studies of factors inducing cellular pH response.

## Results

### Fluorescence pH titration curves for the non-invasive measurement of pH in organelles within living yeast cells

The full potential of the presented approach was achieved by selecting probes with suitable spectral properties and well-defined titration buffers. “Permeant” P-buffer for calibration curve measurement was designed to equilibrate the pH of FP-tagged organelles to external buffer pH. This effect will henceforth be termed “permeation” and should not be confused with the more aggressive effects of the permeabilizing and invasive controls (see File S1). Conversely, “neutral” N-buffer used for cell imaging and pH measurement allowed yeast cells to sustain pHi homeostasis and provided a near-native environment during data acquisition. Putative ammonium exporter Ato1p was chosen as target protein because its transmembrane localization [9] allowed the monitoring of pH in areas where ammonia-induced pH changes are more likely to be detected: Ato1p has a predicted transmembrane C-terminal domain facing the peripheral cytoplasm adjacent to the inner face of the plasma membrane (see File S1). Yeast codon-optimized ecliptic variants yEGFP1 [10] and yEGFP3 [11] were selected as pH indicators.

**Figure 1:**
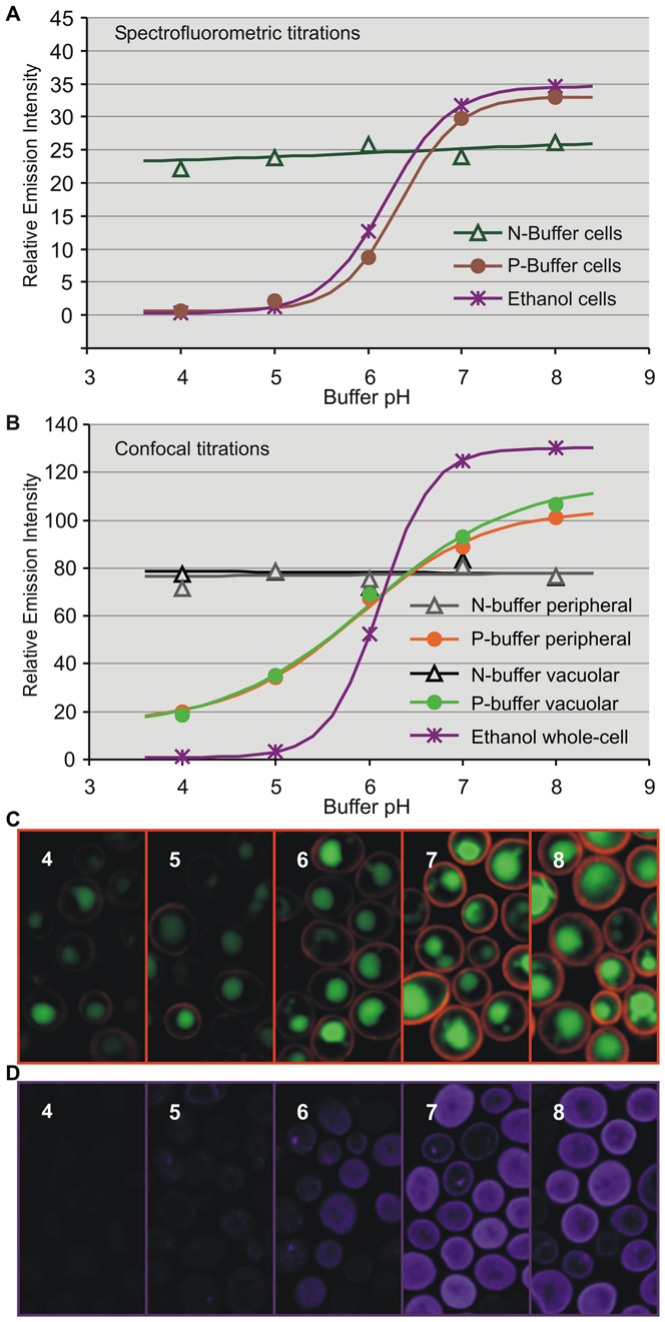
Spectrofluorometer and confocal intensity titrations. Emission intensity (unitless) versus pH titration curves from Ato1p-yEGFP1 fresh cells (A) or individual organelles within fresh cells (B) suspended in N or P-buffers of pH 4–8. Spectrofluorometric analyses and confocal scans were respectively completed within 5 and 3 min after buffer suspension. Treatments with high-dose ethanol (60%) for 2 min before N-buffer suspension were included as invasive controls. Examination of the titration curves (A and B) implied a connection between the sigmoid slope and the degree of cell permeation, but only confocal titration curves (B) evidenced the altered response of ethanol-treated cells. Fluorescent signal for the confocal titrations was acquired from discrete ROIs in the confocal stacks (C and D). Confocal stack images of P-buffer cells with peripheral and vacuolar ROIs (C). Confocal stack images of ethanol-treated cells with whole-cell ROIs (D) revealing the effects of high ethanol doses on cell and organelle integrity. Images were digitally colored to depict ROI sampling.

Emission spectra of *S. cerevisiae* BY4742 expressing *ATO1* C-terminally tagged with either yEGFP1 or yEGFP3 were recorded with a spectrofluorometer using different pH buffers as well as permeabilizing and invasive treatments to calculate intensity titration curves for the optimization of buffer composition (Figures 1, *A* and S1). Ato1p-yEGFP1 cells in N-buffer retained constant intensities within the pH and time range needed for our measurements, resulting in horizontal titration curves. In contrast, Ato1p-yEGFP1 fresh cells in P-buffer responded to pH in a Boltzmann sigmoidal pattern comparable to those exhibited by positive permeabilizing and invasive controls (Figure S1). Results indicated that cells suspended in N-buffer were not influenced by external pH changes, while cells suspended in P-buffer were efficiently permeated (for detailed buffer composition, see Figure S2).

Confocal laser scanning microscopy was employed to complement spectrofluorometric bulk cell analyses with intensity titrations from separate subcellular regions of interest (ROIs) (Figure 1, B). Fluorescence intensities were collected from vacuoles (vacuolar ROIs) and the peripheral cytoplasm adjacent to the plasma membrane (peripheral ROIs) (Figure 1, *C*). Unlike spectrofluorometric titrations (Figure 1, *A*), confocal intensity titrations were able to reveal the expected adverse effects of invasive permeabilizing methods, which can seriously compromise the integrity of vacuoles and probably other organelles (Figure 1, *D*).

### Wavelength emission shifts from ratiometric pHluorin and mCherry are highly robust to low-intensity artifacts and more photostable than pHluorin dual intensity ratios

Titrations based on ecliptic intensity measurements provided qualitative information on the pH of individual organelles, but their susceptibility to intensity interference rendered them unsuitable for precise pHi quantification, especially in the case of weak fluorescence associated with fine subcellular structures (such as plasma membranes), faint FPs or target gene-FP fusions with low expression. In order to perform more robust measurements it was necessary to introduce ratiometric fluorophores with distinct photostable wavelength changes and pKa values as close to physiological levels as possible: yE^2^GFP, a novel yeast codon-optimized version of E^2^GFP designed to display ratiometric spectral shifts in both excitation and emission [4]; mCherry, a monomeric variant of DsRed from *Discosoma sp.* [12] with excitation/emission ratiometric activity [5] and ratiometric pHluorin [3], a widespread GFP mutant known for its pH-dependent excitation ratio changes (not to be confused with any of the ecliptic pHluorin variants).

*S. cerevisiae* giant colonies were grown on GMA solid medium to study pH changes which characterize their 1^st^ acidic, alkali and 2^nd^ acidic developmental phases [6], [8]. Putative ammonium exporter Ato1p was the main target protein because of its high expression and its reported involvement in the alkali and 2^nd^ acidic phases [7], [9]. In addition, monocarboxylate proton symporter Jen1p was also chosen as target protein because of its transmembrane localization [13] and its higher expression during the 1^st^ acidic phase. Both Ato1p and Jen1p have predicted cytosolic C-termini (see File S1).

**Figure 2:**
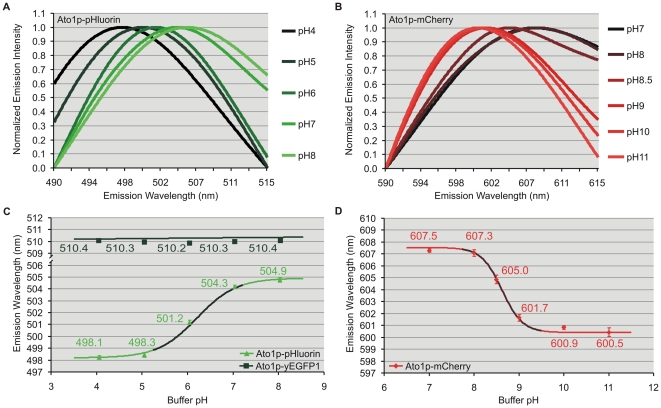
Confocal wavelength titrations. Emission spectra (nm) at different pH from peripheral ROIs within Ato1p-pHluorin (A) and Ato1p-mCherry (B) yeast cells suspended in P-buffers of pH 4–11. Emission intensities were normalized to better show peak shifts. Emission wavelength versus pH titration curves from Ato1p-pHluorin (C) or Ato1p-mCherry (D) peripheral ROIs. Both fluorophores exhibited pH-dependent wavelength shifts indicative of ratiometry by emission at the given pH ranges. Ecliptic yEGFP1 maintained a constant wavelength as expected (C). Representative values were averaged from independent subsets of 10 cells with 20 subcellular ROIs each. Error bars indicate the standard error. The darker areas within the sigmoidal titration curves represent their quantitation limits.

Confocal spectral analyses (Leica TCS SP2/AOBS) of BY4742 cells expressing *ATO1* or *JEN1* with C-terminally fused mCherry, yE^2^GFP, pHluorin or yEGFP1 and suspended in P-buffers were performed to build wavelength titration curves. The low quantum yield of Jen1p-yE^2^GFP required additional ultra-fast spectral detection (Nikon A1) for signal enhancement with minimal photobleaching. CFM scans under rigorously controlled experimental conditions established pH-dependent emission shifts for the three ratiometric fluorophores and verified the absence of wavelength change for ecliptic yEGFP1, which served as a ratiometric negative control (Figures 2 and S3). The emission maxima of yE^2^GFP (λ_ex_ 488 nm) red shifted between pH 5 to 8, with an apparent pKa value of 6.1 (Figure S3). Ratiometric pHluorin exhibited the conventional pH-dependent dual excitation peaks at 395 and 475 nm; but in addition, the more sensitive CFM approach detected an unprecedented red shift of its emission maxima (λ_ex_ 476 nm) under more alkaline pH conditions, with a calculated pKa value of 6.2 (Figure 2, *A* and *C*). In the case of mCherry, its emission maxima (λ_ex_ 543 nm) underwent a blue shift with a pKa of 8.6 (Figure 2, *B* and *D*). The pKa and quantitation limits of the titration curves indicate the pH range and minimum wavelength change from which each FP can produce accurate pH estimates. Ratiometric pHluorin was suitable for pH quantification between 5.2 and 7.3; while mCherry worked optimally between pH 7.7 and 9.5.

**Figure 3:**
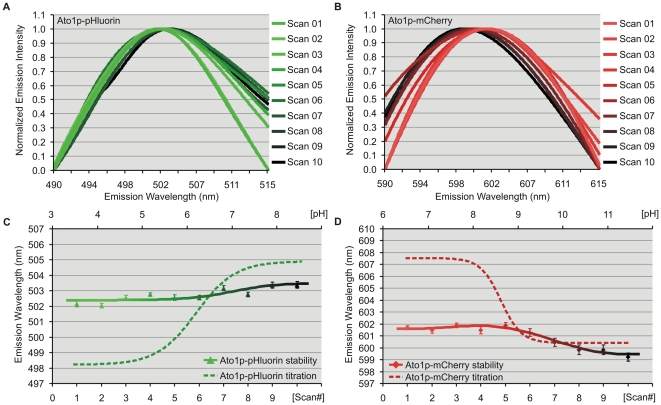
Confocal wavelength stability tests. Emission spectra (nm) of sequential scans from peripheral ROIs within Ato1p-pHluorin (A) and Ato1p-mCherry (B) yeast cells under constant pH conditions. Emission intensities were normalized to better show peak shifts. Wavelength stability curves (solid lines) track peak wavelength maxima throughout 10 successive confocal spectral scans, evidencing an absence of statistically significant changes in Ato1p-pHluorin peripheral wavelength (C) and in the case of Ato1p-mCherry (D), modest fluctuations after the seventh scan. CFM wavelength titrations (dashed lines) projected over the additional top x-axis (pH) are included for comparison to illustrate the higher extent of the pH-induced emission shifts for both ratiometric FPs.

Spectrofluorometric analysis was used to verify the observed pHluorin and mCherry wavelength shifts (Figure S4, *A* and *B*) as well as their stability throughout a series of sequential scans (Figure S4, *C* and *D*). CFM sequential scans were also performed to assess the possibility of spectral artifacts under excessively prolonged experimental conditions (Figure 3, *A*–*D*). After 30 minutes under laser excitation, the fluorescent signal from peripheral ROIs with Ato1p-pHluorin and Ato1p-mCherry showed predictable signs of intensity photobleaching (81% and 52% respectively), but remarkably, they maintained stable emission wavelengths over six-fold longer than the time required for our wavelength-based measurements. Observed fluctuations after the seventh scan remained insignificant in comparison to the full extent of the pH-induced wavelength shifts (Figures 2 and 3). The occurrence of red (pHluorin) and blue (mCherry) shifts under the same pH conditions further disproved the possibility of spectral distortions; since it is highly unlikely such artifacts would be spectrally divergent. All spectrofluorometric and CFM spectral data from our ratiometric FP constructs were systematically fitted to the sum of two or three peaks (see Materials and Methods) to assess the absence of relevant sub-spectral peaks which could interfere with the pH-dependent wavelength shifts (Figures 2, 3 and S4).

**Figure 4:**
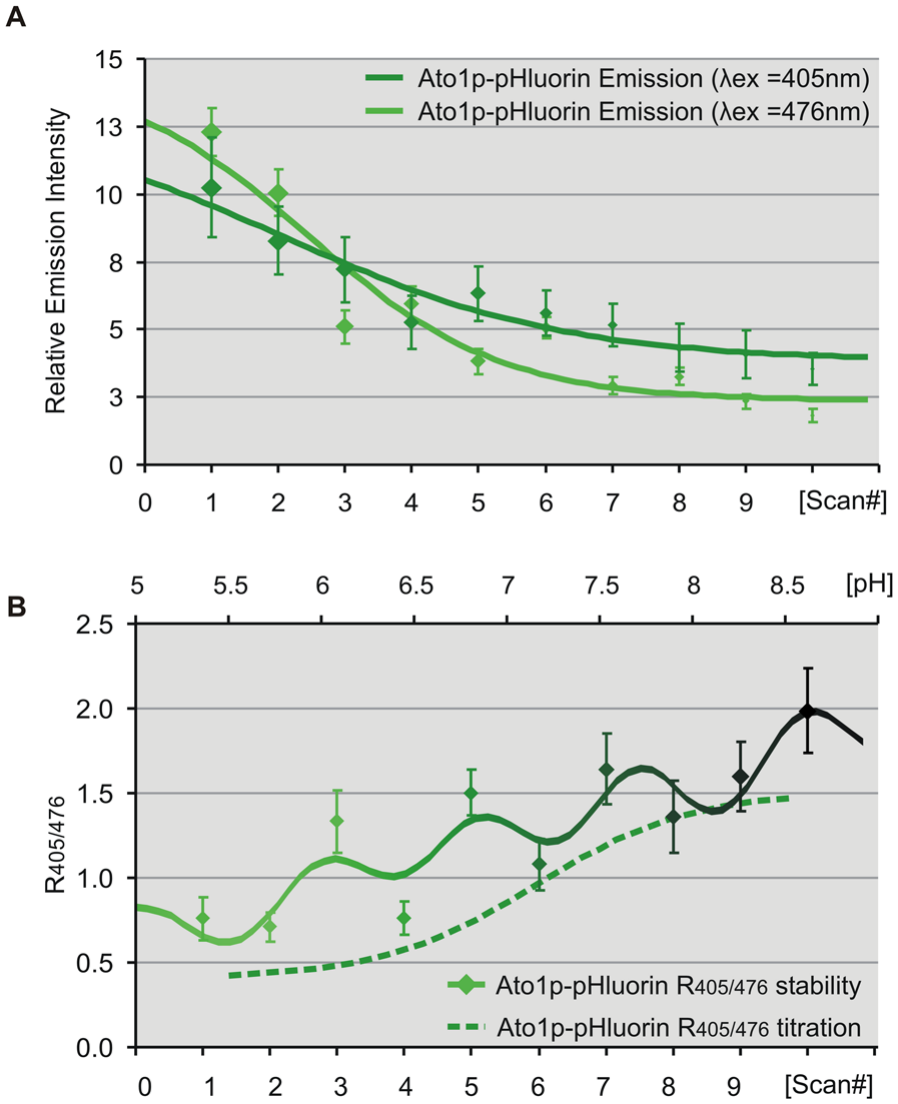
Confocal dual ratio stability tests. (A) Emission intensity (unitless) of sequential scans from peripheral ROIs within Ato1p-pHluorin yeast cells under excitation at 405 and 476 nm and constant pH conditions. (B) Dual ratio stability curves (solid line) track the intensity ratio (R_405/476_) throughout 10 successive confocal spectral scans, evidencing statistically significant changes in Ato1p-pHluorin peripheral ratio after the third scan. Dual ratio titrations (dashed line) projected over the additional top x-axis (pH) illustrate that pH-unrelated ratio fluctuations rapidly exceeded the extent of the pH-induced shifts.

In order to demonstrate the advantages of our wavelength-based approach as opposed to more conventional intensity-based fluorometry, we performed parallel stability tests under comparable photobleaching conditions using the dual excitation peaks of ratiometric pHluorin (Figure 4). Ato1p-pHluorin emission intensity maxima from peripheral ROIs (λ_ex_ 405 and 476 nm) were recorded to calculate intensity ratio (R_405/476_) stability during the sequential scans. Both intensity peaks underwent photobleaching but at differing rates and extent: The intensity maxima of the λ_ex_ 405 peak decreased by 66% with a linear slope of 0.64, while the λ_ex_ 476 peak, despite being the brightest, decreased with a steeper slope of 1.1 by 85% (Figure 4, *A*). These intensity changes altered relative signal-to-noise ratios and the separate effect of autofluorescence over each emission peak, resulting in severe R_405/476_ fluctuations which unlike our wavelength measurements; rapidly exceeded the extent of the pH-induced shifts (Figure 4, *B*).

### In vivo monitoring of pHi changes in individual cells during different stages of yeast colony development

**Figure 5:**
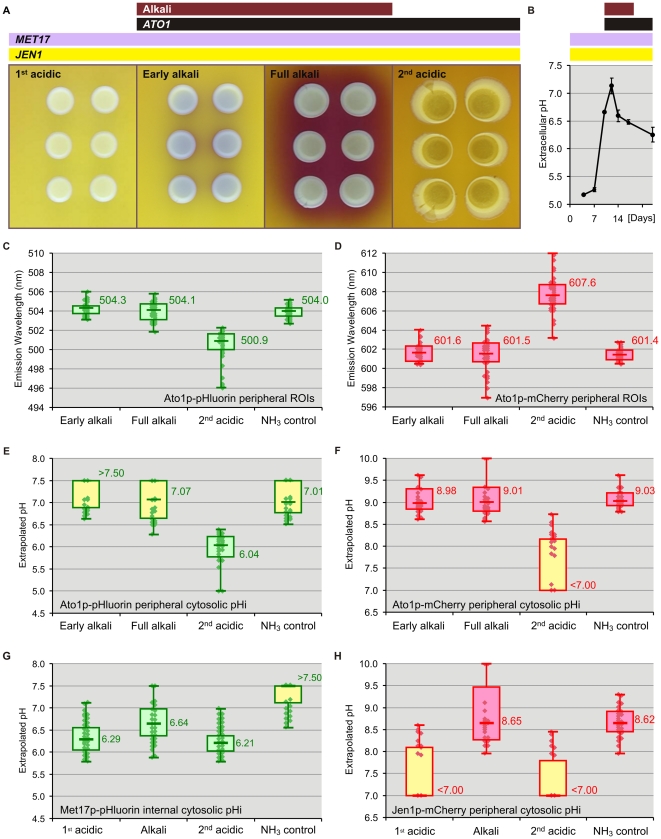
Confocal wavelength-based pH quantifications. Developmental phases of yeast giant colonies growing on GMA plates with BKP pH indicator (A) and extracellular pH changes in the colony surroundings (B). Timings of *ATO1*, *MET17* and *JEN1* gene expression (black, violet and yellow bars) and the ammonia-producing alkali phase (purple bar) are indicated above. Emission wavelength box plots from peripheral ROIs within Ato1p-pHluorin (C) and Ato1p-mCherry cells (D) suspended in N-buffer of pH 6 under near-native conditions. CFM scans were performed during 1^st^ acidic, alkali and 2^nd^ acidic phases. 2^nd^ acidic cells treated with 100 mM NH_3_ served as an alkalinization positive control. Each sample subset was selected to represent cell variability within the colony and contained a total of 30 subcellular ROIs from 3 independent biological repeats. Box plots indicate median, quartiles, extremes and individual values within each subset. Titration-based pH box plots of Ato1p-pHluorin (E) Ato1p-mCherry (F) Met17p-pHluorin (G) and Jen1p-mCherry (H) extrapolated from wavelength data. The effective range of the extrapolations was strictly defined by the pKa and quantitation limits of the titrations. Extrapolations from wavelength populations displaying out-of-range interquartile or medians (yellow box plots) must therefore be treated as non-quantitative and are included for comparison.

Yeast colonies pass through distinct developmental phases characterized by changes in external pH and ammonia production [6], [7], [8]. The wavelength-based measurements from discrete compartments enabled the non-invasive intracellular quantification of actual physiological pH changes in cells taken from colonies at different stages of growth as well as cells exposed to volatile ammonia (NH_3_). Three different target proteins were used for this purpose: The first one, Ato1p, is a transmembrane protein with a cytosolic C-terminus whose expression is triggered by NH_3_ at the beginning of the alkali phase and maintained during the 2^nd^ acidic phase. The other targets are transmembrane protein Jen1p, also with a cytosolic C-terminus, and cytoplasmic protein Met17p. The expression of both Jen1p and Met17p begins at the 1^st^ acidic phase, long before ammonia production, and is maintained throughout the alkali and 2^nd^ acidic phases of colony development (Figure 5). Ato1p-mCherry, Ato1p-pHluorin and Jen1p-mCherry constructs allowed us to monitor cytosolic pH in the periphery of the plasma membrane; while the Met17p-pHluorin construct provided information on the pH from internal regions of the cytoplasm.

**Figure 6:**
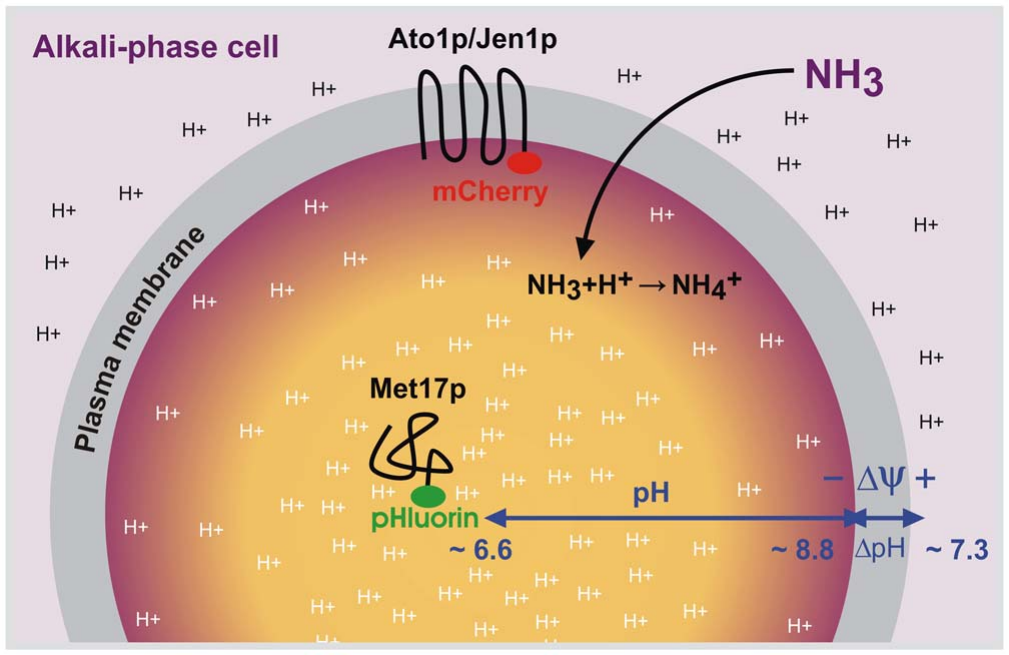
Model of cellular pH gradients. Schematic diagram illustrating intracellular pH gradients within yeast cells from alkali-phase colonies. Measurements with Ato1p-pHluorin, Ato1p-mCherry, Jen1p-mCherry and Met17p-pHluorin fusion proteins provided evidence for the existence of sharp intracellular pH gradients between peripheral (∼8.8) and internal (∼6.6) cytoplasmic regions during the ammonia-producing alkali phase. In contrast to protonated ammonium (NH_4_^+^), volatile ammonia (NH_3_) enters the cell by simple diffusion through the plasma membrane. High cytosolic pH values near the plasma membrane allow for the maintenance of high transmembrane electrochemical potential (Δψ) based on H^+^ gradient (ΔpH) under conditions of relatively high extracellular pH (∼7.3).

Cells from Ato1p-mCherry, Ato1p-pHluorin, Jen1p-mCherry and Met17p-pHluorin yeast colonies occurring in different developmental phases were harvested and suspended in pH 6 N-buffer for immediate CFM analysis (Figure 5, *A*). Additional suspensions of 2^nd^-acidic cells were treated with high-dose NH_3_ (100 mM) for 9 minutes before measurements and analyzed as alkalinization positive controls. The timing of the phase transitions was monitored with control GMA plates containing pH indicator bromocresol purple (BKP) (pKa 6.3). Fluorescent signals from peripheral ROIs within both mCherry- and pHluorin-tagged strains underwent large changes in emission wavelength during the alkali/acid transition (Figure 5, *C* and *D*). One-way ANOVA and Tukey's post-hoc tests (α 0.05) of our Ato1p and Jen1p peripheral ROI data confirmed very significant differences (P<0.001) between alkali and acidic populations, while NH_3_ controls were comparable to alkali populations. Median, interquartiles and extreme values from the box plots do not represent measurement error, but the variability among the measurements from the 30 individual subcellular ROIs which comprise each sample cell population. Wavelength-based pHi extrapolations from Ato1p-mCherry and Jen1p-mCherry peripheral ROIs during the alkali phase or NH_3_ treatment (Figure 5, *F* and *H*) implied a substantial cytosolic alkalinization near the plasma membrane while those from 2^nd^-acidic Ato1p-pHluorin peripheral ROIs (Figure 5, *E*) indicated usual pHi in yeast cells under acidic conditions [6], [8], [14], [15], [16]; evidencing a correlation between peripheral cytosolic pH and the pH surrounding the colonies (Figure 5, *B*). In comparison, Met17p-pHluorin cells exhibited relatively smaller changes: extrapolations from Met17p-pHluorin cytosolic ROIs suggested more stable pHi conditions in deeper cytosolic regions throughout all stages of colony development (Figures 5, *G* and 6). In all experiments, samples exposed to high-dose NH_3_ exhibited significantly lower wavelength variability than untreated alkali populations due to the treatment's homogenizing effect on all cells. The physiological relevance of this finding is discussed below.

Comparative spectrofluorometric pH extrapolations with Ato1p-pHluorin cells and based on pHluorin's dual intensity ratios showed much lower pH fluctuations in the peripheral cytoplasm (Figure S5, *A*–*B*). Alkalinization was only noticeable after artificial NH_3_ treatment. These results were in opposition to the CFM wavelength-based measurements with identical cells, but strongly resembled the Met17p-pHluorin deep cytosolic results.

## Discussion

### The importance of confocal microscopy in the control of cell integrity and the determination of subcellular pH under near-native conditions

Fluorescence pH titration curves and pH determination are routinely obtained by cuvette or microplate spectrofluorometry of FP-expressing cells. Such averaged bulk cell measurements provide useful preliminary information on the behavior of the fluorophores and on cellular pHi values, but like most in vivo fluorometric techniques, they are greatly hindered by low signal-to-noise ratios and lack the single-cell resolution required for pH analyses within living cell compartments.

We instead resorted to confocal fluorescence microscopy in order to measure pH at the subcellular level, improve signal detection and retrieve faint FPs with useful pKa values as for example yEGFP3 and yE^2^GFP. More importantly, confocal imaging allowed for the selective sampling of viable cells, and provided the means to guarantee the near-native state of cells suspended in N-buffer for non-invasive in situ pHi estimations as well as the efficient permeation of plasma and vacuolar membranes during calibrations with P-buffer.

Fluorescence microscopy imaging of pH-sensitive FPs like ecliptic and ratiometric pHluorin is widely used in various model organisms and research fields, such as the study of intracellular pH [14], [17], [18], [19], [20] or synaptic vesicle endocytosis [21], [22], [23]. Additionally, the advantages of ROI analysis and in situ pHi calibrations have been accepted for years [24], [25], [26]. However, and in spite of that, a number of recent studies on intracellular pH remain focused on spectrofluorometry [16], [27], [28], [29]. These studies commonly complement their pH measurements with fluorescent microscopy imaging to confirm fluorophore localization prior to analysis, but are unable to ascertain cell fitness, subcellular integrity or organelle specificity of the signal during and after data acquisition. As a consequence, spectrofluorometric measurements are susceptible to interference by common pH-unrelated fluorescent events such as increased fluorescence from dying cells or internalization/degradation of the fluorescent protein in vacuoles and lysosomes. These altered fluorescent signals may not account for a large percentage within the bulk cell suspension, but their intensity is often stronger than the targeted FP fusion proteins and can contribute very significantly to the overall fluorescence measured [30], [31], [32]. Moreover, such internalized and degraded fluorescent signals cannot be eliminated by background subtraction since they are not part of the cell autofluorescence background but a fraction of the total fluorophore signal. They can however be easily isolated by subcellular ROI analysis (see Materials and Methods).

Our spectrofluorometric dual ratio-based pHi extrapolations with Ato1p-pHluorin cells provided empirical evidence of such problems: The lower pHi values allegedly measured in the peripheral cytoplasm of cells from alkali-phase colonies (Figure S5) can be explained by the fact that data were not obtained from peripheral ROIs, but from averaged bulk cell suspensions. Spectrofluorometric results thus resembled the CFM readings from internal regions of the cytoplasm (Figure 5, *G*) because the subtle peripheral fluorescence from Ato1p-pHluorin cells was masked by brighter subcellular signals (possibly vacuoles) and consequently lost in the spectrofluorometric bulk average. This interpretation was clearly supported by the spectrofluorometric data obtained from NH_3_-treated samples, the only ones to exhibit alkalinization comparable to CFM readings: Unlike gradually produced low amounts of NH_3_ in cells from alkali-phase colonies, which mainly affects the peripheral cytoplasm, the exogenous application of high-dose NH_3_ leads to a sharp increase of cytosolic pH throughout the whole cell (documented also by CFM of Met17-pHluorin samples, Figure 5, *G*). It has been suggested that NH_3_ could even enter and alkalinize cell vacuoles [33]. Such whole-cell alkalinization is stronger and more wide-reaching than the effect of naturally produced ammonia, and therefore more detectable by bulk spectrofluorometry.

### Wavelength instead of intensity-based measurements allow for more accurate physiological pHi quantifications

Several methods have been successfully employed to increase the fluorescent signal of ecliptic and ratiometric fluorophores. In the particular case of pHluorin, some involve the use of constitutive or inducible over-expression [18], [29], [34], [35] while others rely on the development of enhanced pHluorin mutants [17], [20], [25]. Even though intensity enhancement helps optimize signal-to-noise ratios in a similar way to our CFM approach, it is not applicable to all situations: the study of intracellular physiological events usually requires the use of non-constitutive target genes like *ATO1*, whose expression levels can decrease drastically during certain physiological stages and lead to low-signal artifacts.

Titration curves based on the emission wavelength shifts from pH-dependent ratiometric fluorophores eliminated many problems associated with signal intensity loss and yielded sufficiently accurate pHi estimates for true quantitative analysis. Wavelength stability tests with mCherry and pHluorin confirmed the absence of significant spectral distortions due to the experimental setup, and together with pHluorin's dual intensity ratio stability tests, they corroborated that ratiometry by emission is considerably more resistant to photobleaching and pH-unrelated low-signal artifacts than intensity-based pH sensitivity. An example of the above mentioned artifacts is provided by a recent study based on ratiometric pHluorin [27], in which titration curves for cytosolic and mitochondrial pHluorin showed an unexplained but very significant difference in their apparent pKa values (>1.25 units). Theoretically, titrations performed with the same fluorophore can differ in sigmoid height but should have comparable dissociation constants. As illustrated by Ato1p-pHluorin dual ratio stability tests, such pKa discrepancies can result from imbalances in the pH response of the peak intensity ratio, which may occur when the intensity of one of the emission peaks falls below autofluorescence levels, thus becoming undetectable and virtually unresponsive to variations in pH.

Each of the ratiometric FPs in our study were intended to cover a limited pH range according to their individual quantitation limits and pKa values, but in combination they contributed to expand the scope of quantification toward more alkali physiological pH conditions. Ratiometric pHluorin has a dual ratio-based pKa of 7.2 and an effective quantitation limits between pH 5.5 and 7.5 [24]; but there are many examples of physiological pHi values beyond these limits [2], [15], [16], [36], [37]. Previous studies have stressed the critical importance of pKa and quantification limits for the usefulness of pHluorin [15], [17] and employ alternative ratiometric dyes or non-ratiometric FPs with more appropriate pKa values when measuring extreme pH values [16], [24], [28]. In spite of all these suggestions, a number of out-of-range pHi extrapolations with ratiometric pHluorin have been performed in peroxisomes [35], [38] mitochondria [27] or vacuoles [25]. These organelles are believed to have pH values around 8.2 [15], 8.0 [2] and 4.8 [16], respectively, which fall outside the quantitation limits of ratiometric pHluorin. The resulting loss of quantitative accuracy will render any pH estimate approximate. To illustrate these problems we included out-of-range pHluorin extrapolations from cells in alkali-phase colonies: The significant decrease in wavelength variability, particularly toward longer wavelengths, observed in Ato1p-pHluorin alkali-phase colonies (Figure 5, *C*), suggested that the upper quantitation limit of pHluorin's alkaline-dependent red shift was reached in a substantial part of the sampled subcellular population. Ignoring this fact would lead to biased pH extrapolations. These pKa and quantitation restrictions apply to all ecliptic and ratiometric fluorescent pH probes. The present study also recommends ratiometric mCherry (pKa 8.6) as a valid complement to pHluorin.

### Dynamic pH changes and subcellular pH gradients within yeast cells from colonies at different development stages

The CFM protocol succeeded in quantifying pH within living cells and intracellular compartments while minimizing sample manipulations likely to disturb cell metabolic functions. As a result we were able to perform non-invasive studies of physiological pHi under near-native conditions.

Yeast colonies passing through distinct developmental phases change the pH of their surroundings from acidic to alkaline and vice versa [9]. During the 1^st^ acidic phase most of the available nutrients are consumed. As colonies begin to starve the level of intracellular stress increases and the first volatile ammonia molecules are released into the extracellular space. Ammonia production quickly intensifies via a feedback mechanism resembling quorum sensing [6] and triggers the beginning of an alkali phase characterized by extensive metabolic reprogramming [39]. Volatile ammonia has been shown as an important signaling molecule in the development cycle of other organisms such as *Dictyostelium discoideum* and in mammalian neurons [9], [33], [40]. Unprotonated NH_3_ functions as the active molecule in yeast colony signaling whereas neither protonated ammonium (NH_4_^+^) nor alkali itself have signaling function [6]. In contrast to NH_4_^+^, volatile NH_3_ penetrates into yeast cells by simple diffusion through the plasma membrane [41]. The molecular mechanism of NH_3_ signaling is currently unknown, but earlier studies on *D. discoideum* have hinted that NH_3_ could enter the acidic compartments (vacuoles) and become there protonated [33]. The resulting dissipation of the proton gradient across the vacuolar membrane would then lead to changes in cell behavior.

The results from yeast giant colony experiments are of particular biological significance. They evidence with a high degree of certainty the existence of dynamic pHi changes throughout colony development as well as sharp pHi gradients related to the distance from the membrane within cells from colonies occurring in the ammonia-producing alkali phase. Given that *JEN1* expression and function [13] are not directly related to NH_3_ signaling, peripheral pH extrapolated from Jen1p-mCherry cells from acidic- and alkali-phase colonies (Figure 5, *H*) corresponded to that of Ato1p-FP cells. This finding supports the conclusion that the observed changes in peripheral ROI fluorescence were most likely induced by pH rather than being, for example, a side effect of NH_3_ on the protein conformation of FP-tagged putative ammonium exporter Ato1p. Peripheral cytosolic pHi reached values of about 8.8 during the alkali phase, while internal cytosolic pH fluctuated around 6.6, differing only slightly from pH values of acidic-phase colonies (6.3, approximately). Alkaline pHi values measured in the cytoplasm adjacent to the inner face of the plasma membrane (where volatile ammonia enters the cells) could explain the high transmembrane electrochemical potential measured in cells from alkali-phase colonies with extracellular pH values above 7 [7], [42]. On the other hand, lower pHi conditions in deeper cytosolic regions would facilitate various metabolic reactions taking place in the cytoplasm (Figure 6). The decreased pH variability in deep cytosol (Met17p-pHluorin ROI population) also suggests that cells within alkali-phase colonies have somehow to regulate pH and sustain homeostasis in specific parts of the cytoplasm during ammonia production. In contrast, the cells were not able to adapt internal cytosolic pH immediately after application of high-dose NH_3_ applied from an artificial source.

The approach developed in this study opens new possibilities in the field of fluorometry: Bulk or single-cell analyses obtained by conventional spectrofluorometry, FACS flow cytometry or CE-LIF [43], [44] may be easily enhanced with CFM and ratiometric FPs to provide higher fluorescence sensitivity, non-invasive subcellular data and quantitative information as well as increase tissue penetration depth and analytical resolution; a particularly interesting feature for in situ measurements in multicellular organized structures, from microbial colonies and biofilms to plant and animal tissues [24].

## Materials and Methods

### Yeast strains and plasmids

Ato1p-yEGFP1, Ato1p-yEGFP3, Ato1p-pHuorin, Ato1p-mCherry, Jen1p-mCherry, Jen1p-yE^2^GFP and Met17p-pHluorin strains were derived from the *S. cerevisiae* BY4742 strain (*MATα*, *his3Δ*, *leu2Δ0*, *lys2Δ0*, *ura3Δ0*) obtained from the EUROSCARF collection. All mutant strains retained the phenotypic characteristics of their parental lines.

### Media and growth conditions

*S. cerevisiae* giant colonies [9] for phenotypic or fluorescence analysis were grown at 28°C on GMA plates (1% yeast extract, 3% glycerol, 2% agar, 10 mM CaCl_2_*2H_2_O) with 6 colonies per plate. Colonies generally reached the alkali phase at approximately day 10 of development, and the 2^nd^ acidic within the next 7 days. In experiments where colony pH had to be monitored, duplicate sets of GMA plates were supplied with 0.01% BKP and incubated in parallel. BKP changes from yellow to purple between pH 5.2 and 6.8.

### Construction of the strains

BY-Ato1p-yEGFP1 and BY-Ato1p-yEGFP3 strains were constructed as previously described [45]. BY-Ato1p-pHluorin and BY-Met17p-pHluorin strains were kindly provided by A. Holoubek. The strains carry C-terminal GFP variant tags. BY-Ato1p-mCherry and BY-Jen1p-mCherry strains carrying monomeric red fluorescent mCherry [12] C-terminally fused to either Ato1p or Jen1p were developed from BY4742 cells according to standard procedures [46], [47] (see File S1 and Figure S6). The construction of BY-Jen1p-yE^2^GFP strains was preceded by the preparation of yE^2^GFP, a new yeast codon-optimized variant of E^2^GFP (F64L/S65T/T203Y) [4] with the QuikChange Site-Directed Mutagenesis kit (Stratagene, La Jolla, CA). Target protein Jen1p was C-terminally tagged with yE^2^GFP at the endogenous locus. A detailed description of strain construction and selection is given in the Supporting Information.

### N- and P-buffers

N-buffer used for cell imaging or control titrations with nonpermeated cells contained equimolar (50 mM) mixtures of biological buffers HEPES and MES or HEPES, Tris and CHES (N-Cyclohexyl-2-aminoethanesulfonic acid) depending on the pH ranges of interest (see Figure S2). Final pH values were adjusted between 4.0 and 11.0 with HCl or NaOH. The addition of 50 mM KCl and 50 mM NaCl stabilized cells in long-term measurements (>30 minutes) but was not needed within the usual 5-minute span. P-buffer used for in vivo calibrations had the same composition as N-buffer in addition to 10 mM sodium azide, an inhibitor which decreases H^+^-ATPase proton pump activity through intracellular ATP depletion; and 0.2 M ammonium acetate, an electrolyte which reportedly equilibrates internal and buffer pH across plasma and vacuolar membranes [48], [49]. Several intermediate buffers were set aside during buffer optimization with the purpose of evaluating the role of each solution component. Sequentially, S-Buffer was N-buffer with KCl and NaCl; H-Buffer was S-Buffer with ammonium acetate, and P-buffer was H-Buffer with sodium azide. An additional treatment before P-Buffer suspension with 10 µM nigericin (Sigma-Aldrich, Munich, Germany), a K^+^/H^+^ ionophore, was included as a positive permeabilizing control. Figure S1 depicts the comparative performance of all buffer treatments.

### Fluorescence spectral acquisition

For in vitro spectrofluorometric titrations, 800 µl yeast suspension aliquots (OD 0.5) were placed in 2.5 ml cuvettes under constant stirring and measured with a Horiba Jobin Yvon FluoroMax-P spectrofluorometer. Emission spectra were collected at 490–560 nm (λ_ex_ 475 nm) for all GFP variants, and at 490–620 nm (λ_ex_ 525 nm) for mCherry. For dual ratio spectrofluorometric measurements with pHluorin, emission spectra were collected at 505–535 nm upon excitation at 470 and 490 nm (±15 nm). In all cases, blank scans of BY4742 suspensions were used to subtract non-specific background signal. Long pass filters (Omega Optical, Brattleboro, VT) were employed to block excitation light when necessary. Cell lysate was prepared by glass-bead lysis as previously described [7]. For in situ confocal microscopy titrations 0.5 µl cell suspension aliquots were pipetted on coverslips and immobilized with 0.8% agarose films which were prepared with the same buffers used for each pH point to prevent osmotic or pH imbalance. Confocal spectral scans were normally performed with a Leica TCS SP2/AOBS (Leica Microsystems, Heidelberg, Germany) or, where indicated, with a Nikon A1 (Nikon, Tokyo, Japan). Laser dose, acquisition parameters and excitation light exposure times were adjusted with the brightest sample to optimize signal-to-noise ratios, minimize photobleaching and maintained constant throughout each pH titration series. Acquisition optimization lessened the weight of pH-unrelated intensity fluctuations in dim samples. Emission profiles were recorded at 493–543 nm (Ar laser, λ_ex_ 488 nm) for yeast strains expressing yEGFP1, yEGFP3 or yE^2^GFP, at 483–533 nm (Ar laser, λ_ex_ 476 nm) for BY-Ato1p-pHuorin lines and at 580–630 nm (HeNe laser, λ_ex_ 543 nm) for mCherry strains. For dual ratio confocal measurements with BY-Ato1p-pHuorin cells, emission spectra were acquired using frame-by-frame sequential excitation with Ar (λ_ex_ 476 nm) and blue diode (λ_ex_ 405 nm) laser lines in order to calculate pHluorin's intensity ratio (R_405/476_).

### Single-cell sampling and subcellular analysis

Before confocal scanning, areas from the field of view were selected to represent the variability within the cell population. Unless otherwise specified, all representative data were averaged from 3 independent biological repeats, each comprising 20 organellar regions from 10 individual cells. Single cells were randomly sorted from each repeat, excluding only the apoptotic or disrupted and avoiding bias towards the brightest. Spectral stacks were processed using LCS LITE software version 2.61 (Leica Microsystems, Mannheim, Germany) or NIS ELEMENTS AR 3.00 software (Laboratory Imaging, Prague, Czech Republic). Systematic delineation of regions of interest (ROIs) made it possible to isolate emission spectra from separate and specific organelles of interest, further improve signal-to-noise ratios and evaluate cell viability among titration points.

### Peak profile and curve fitting

Choosing a proper nonlinear regression model was essential to extract information from the shape of the titration curves. FITYK curve fitting freeware [50] and FINDGRAPH software (Uniphiz Lab, Vancouver, Canada) were used to calculate the best fitting equations with minimal standard error. Emission spectra profiles under changing pH were generally fitted to sums of one or two Lorentzian peak functions plus a Gaussian y(x) = A*[[((1−*D*)*(ln2^1/2^))/(C*(*π* ^1/2^))] * [exp(−ln2*((x−*B*)/*C*)^2^)]+[*D*/((π*C)*(1+((x−*B*)/*C*)^2^))]] where y(x) is emission intensity, x is the emission wavelength and *A* through *D* are peak fitting parameters. Peaks and intensity maxima obtained from the emission profiles were plotted against pH to produce intensity and wavelength titrations. Calibration curves of permeated cells typically adopted a sigmoidal shape best fitted to the Boltzmann equation y(x) = (*A*+(*B*−*A*))/(1+exp(−(x−*C*)/*D*) where y(x) is either emission intensity or wavelength, x is the buffer pH, *A* and *B* delimit the extent of the fluorophore response (sigmoid height), *C* is the inflection midpoint of the exponential phase (where pKa equals pH) and *D* defines the slope transition between exponential and plateau phases. The quantitation limits of the sigmoidal curves are given by QL = (10*σ)/*S* where *σ* is the standard deviation of linear regression y-intercepts and *S* is the maximum slope at the inflection midpoint. Control calibrations with neutral buffer or samples exhibiting a limited pH-dependent response were better described by sigmoids with very small slopes or simple linear regression models.

## Supporting Information

Figure S1 **Spectrofluorometer intensity titrations.** Emission intensity (unitless) versus pH titration curves from Ato1p-yEGFP1 fresh cells and cell lysate in calibration buffers of pH 4–8 with increasing permeating capacity (N, S, H and P-buffer). Titration curves of fresh cells (A) and cell lysate (B) after 5 min in suspension. Titration curves of fresh cells (C) and cell lysate (D) after 30 min in suspension. Cell lysis and treatment with high-dose ethanol (60%) before N-buffer suspension were included as invasive controls, while treatment with 10 µM nigericin before P-buffer suspension was a positive permeabilizing control. Examination of the titration curves implied a connection between the sigmoid slope and the degree of cell permeation. The titration curve achieved after sodium azide addition (transition from H to P-Buffer) was comparable to that of cell lysate, nigericin and ethanol controls; suggesting efficient transmembrane H^+^ equilibration.
(TIF)

Figure S2 **Titration buffer composition.** Biological buffers HEPES and MES were employed in all titrations from pH 4 to 8. * For mCherry titrations from pH 6 to 11, MES was replaced with Tris and CHES to cover the higher alkali range. KCl and NaCl stabilized cells under long-term or permeant conditions. The combined interaction of ammonium acetate and sodium azide mediated the permeating capacity of the buffers. Nigericin was only added to the P-buffer solution in the positive permeabilizing controls.
(TIF)

Figure S3 **Confocal wavelength titrations.** Emission wavelength (nm) versus pH titration curves from organellar ROIs within yeast cells suspended in P-buffer of pH 4–11. Strains expressing ratiometric pHluorin, yE^2^GFP and mCherry exhibited pH-dependent wavelength shifts indicative of ratiometry by emission at the given pH ranges. Representative values were averaged from individual subsets of 10 cells with 20 subcellular ROIs each. Error bars indicate the standard error. The darker areas within the sigmoidal titration curves represent their quantitation limits.
(TIF)

Figure S4 **Spectrofluorometer wavelength stability tests.** Emission spectra (nm) at different pH from Ato1p-pHluorin (A) and Ato1p-mCherry (B) fresh cells suspended in P-buffers of pH 4–11. Emission intensities were normalized to better show peak shifts. Wavelength stability curves (solid lines) from Ato1p-pHluorin (C) and Ato1p-mCherry (D) fresh cells illustrate the absence of emission spectral fluctuations throughout 50 successive spectrofluorometric scans under different pH conditions. Preliminary spectrofluorometer wavelength titrations (dashed lines) projected over the additional top x-axis (pH) are included for comparison.
(TIF)

Figure S5 **Spectrofluorometer dual ratio-based pH quantifications.** (A) Dual ratios (R_410/470_) from peripheral ROIs within Ato1p-pHluorin bulk cell populations suspended in N-buffer of pH 6 under near-native conditions. Spectrofluorometric scans under excitation at 410 and 470 nm were performed during alkali and 2^nd^ acidic phases. 2^nd^ acidic cells treated with NH_3_ served as an alkalinization positive control. Each sample subset was constituted by a total of 22 sequential scans from 2 independent biological repeats. Error bars do not indicate variability within the colony but spectrofluorometric measurement standard error. In comparison to the CFM wavelength-based approach, spectrofluorometric dual ratio-based measurements were not sensitive enough to sufficiently detect physiological pHi changes. (B) Titration-based pH extrapolations from Ato1p-pHluorin (R_410/470_) data. The effective range of the extrapolations was strictly defined by the pKa and quantitation limits of the titrations.
(TIF)

Figure S6 **Primer list.** Primer sequences are presented in 5′ to 3′ orientation. PCR primers (A) are shown in lower case. Mutagenic primers (B) contain 5′ and 3′ end sequences homologous to the target gene (lower case) and codon substitutions (bold case) involving 1–2 bp changes (red case). Cassette primers (C) contain 5′ end sequences homologous to the target gene (upper case) and 3′ end sequences homologous to the template plasmid (lower case).
(TIF)

File S1 **Supporting Information.**
(DOC)

## References

[1] Tsien RY (1998). The Green Fluorescent Protein.. Annu Rev Biochem.

[2] Llopis J, McCaffery JM, Miyawaki A, Farquhar MG, Tsien RY (1998). Measurement of cytosolic, mitochondrial, and Golgi pH in single lying cells with green fluorescent proteins.. Proc Natl Acad Sci U S A.

[3] Miesenböck G, De Angelis GDA, Rothman JE (1998). Visualizing secretion and synaptic transmission with pH-sensitive green fluorescent proteins.. Nature (London, U K).

[4] Bizzarri R, Arcangeli C, Arosio D, Ricci F, Faraci P (2006). Development of a novel GFP-based ratiometric excitation and emission pH indicator for intracellular studies.. Biophys J.

[5] Shu X, Shaner NC, Yarbrough CA, Tsien RY, Remington SJ (2006). Novel chromophores and buried charges control color in mFruits.. Biochemistry.

[6] Palková Z, Forstová J (2000). Yeast colonies synchronise their growth and development.. J Cell Sci.

[7] Váchová L, Kučerová H, Devaux F, Ulehlová M, Palková Z (2009). Metabolic diversification of cells during the development of yeast colonies.. Environ Microbiol.

[8] Imai T, Ohno T (1995). Measurement of yeast intracellular pH by image processing and the change it undergoes during growth phase.. J Biotechnol.

[9] Palková Z, Janderová B, Gabriel J, Zikánová B, Pospíšek M (1997). Ammonia mediates communication between yeast colonies.. Nature (London, U K).

[10] Sheff MA, Thorn KS (2004). Optimized cassettes for fluorescent protein tagging in *Saccharomyces cerevisiae*.. Yeast.

[11] Cormack BP, Bertram G, Egerton M, Gow NAR, Falkow S (1997). Yeast-enhanced green fluorescent protein (yEGFP): a reporter of gene expression in *Candida albicans*.. Microbiology.

[12] Shaner NC, Campbell RE, Steinbach PA, Giepmans BN, Palmer AE (2004). Improved monomeric red, orange and yellow fluorescent proteins derived from *Discosoma sp*. red fluorescent protein.. Nat Biotechnol.

[13] Paiva S, Kruckeberg AL, Casal M (2002). Utilization of green fluorescent protein as a marker for studying the expression and turnover of the monocarboxylate permease Jen1p of *Saccharomyces cerevisiae*.. Biochem J.

[14] Karagiannis J, Young PG (2001). Intracellular pH homeostasis during cell-cycle progression and growth state transition in *Schizosaccharomyces pombe*.. J Cell Sci.

[15] Roermund CWT, de Jong M, IJlst L, van Marle J, Dansen TB (2004). The peroxisomal lumen in *Saccharomyces cerevisiae* is alkaline.. J Cell Sci.

[16] Brett CL, Tukaye DN, Mukherjee S, Rao R (2005). The yeast endosomal Na^+^(K^+^)/H^+^ exchanger Nhx1 regulates cellular pH to control vesicle trafficking.. Mol Biol Cell.

[17] Sankaranarayanan S, De Angelis D, Rothman JE, Ryan TA (2000). The use of pHluorins for optical measurements of presynaptic activity.. Biophys J.

[18] Olsen KN, Budde BB, Siegumfeldt H, Rechinger KB, Jakobsen M (2002). Noninvasive measurement of bacterial intracellular pH on a single-cell level with green fluorescent protein and fluorescence ratio imaging microscopy.. Appl Environ Microbiol.

[19] Disbrow GL, Hanover JA, Schlegel R (2005). Endoplasmic reticulum-localized human papillomavirus type 16 E5 protein alters endosomal pH but not trans-Golgi pH.. J Virol.

[20] Mahon MJ (2011). pHluorin2: an enhanced, ratiometric, pH-sensitive green florescent protein.. Adv Biosci Biotechnol.

[21] Ashby MC, Ibaraki K, Henley JM (2004). It's green outside: tracking cell surface proteins with pH-sensitive GFP.. Trends Neurosci.

[22] Granseth B, Odermatt B, Royle SJ, Lagnado L (2007). Clathrin-mediated endocytosis: the physiological mechanism of vesicle retrieval at hippocampal synapses.. J Physiol.

[23] McDonald NA, Henstridge CM, Connolly CN, Irving AJ (2007). Generation and functional characterization of fluorescent, N-terminally tagged CB1 receptor chimeras for live-cell imaging.. Mol Cell Neurosci.

[24] Nehrke K (2006). Intracellular pH measurements *in vivo* using green fluorescent protein variants.. Methods Mol Biol (Totowa, NJ, U S).

[25] Bagar T, Altenbach K, Read ND, Benčina M (2009). Live-cell imaging and measurement of intracellular pH in filamentous fungi using a genetically encoded ratiometric probe.. Eukaryot Cell.

[26] Dechant R, Binda M, Lee SS, Pelet S, Winderickx J (2010). Cytosolic pH is a second messenger for glucose and regulates the PKA pathway through V-ATPase.. EMBO J.

[27] Orij R, Postmus J, Ter Beek A, Brul S, Smits GJ (2009). *In vivo* measurement of cytosolic and mitochondrial pH using a pH-sensitive GFP derivative in *Saccharomyces cerevisiae* reveals a relation between intracellular pH and growth.. Microbiology.

[28] Braun NA, Morgan B, Dick TP, Schwappach B (2010). The yeast CLC protein counteracts vesicular acidification during iron starvation.. J Cell Sci.

[29] Marešová L, Hošková B, Urbánková E, Chaloupka R, Sychrová H (2010). New applications of pHluorin - measuring intracellular pH of prototrophic yeasts and determining changes in the buffering capacity of strains with affected potassium homeostasis.. Yeast.

[30] Plant PJ, Manolson MF, Grinstein S, Demaurex N (1999). Alternative mechanisms of vacuolar acidification in H+-ATPase-deficient yeast.. J Biol Chem.

[31] Bour-Dill C, Marie-Pierre G, Jean-Louis M, Marchal S, Guillemin F (2000). Determination of intracellular organelles implicated in daunorubicin cytoplasmic sequestration in multidrug-resistant MCF-7 cells using fluorescence microscopy image analysis.. Cytometry.

[32] Varlamov O, Somwar R, Cornea A, Kievit P, Grove KL (2010). Single-cell analysis of insulin-regulated fatty acid uptake in adipocytes.. Am J Physiol Endocrinol Metab.

[33] Davies L, Satre M, Martin JB, Gross JD (1993). The target of ammonia action in dictyostelium.. Cell.

[34] Moseyko N, Feldman LJ (2001). Expression of pH-sensitive green fluorescent protein in *Arabidopsis thaliana*.. Plant Cell Environ.

[35] Lasorsa FM, Scarcia P, Erdmann R, Palmieri F, Rottensteiner H (2004). The yeast peroxisomal adenine nucleotide transporter: characterization of two transport modes and involvement in ΔpH formation across peroxisomal membranes.. Biochem J.

[36] Matsuyama S, Llopis J, Deveraux QL, Tsien RY, Reed JC (2000). Changes in intramitochondrial and cytosolic pH: early events that modulate caspase activation during apoptosis.. Nat Cell Biol.

[37] Mukherjee S, Kallay L, Brett CL, Rao R (2006). Mutational analysis of the intramembranous H10 loop of yeast Nhx1 reveals a critical role in ion homoeostasis and vesicle trafficking.. Biochem J.

[38] Jankowski A, Kim JH, Collins RF, Daneman R, Walton P (2001). In situ measurements of the pH of mammalian peroxisomes using the fluorescent protein pHIuorin.. J Biol Chem.

[39] Palková Z, Devaux F, Řičicová M, Mináriková L, Le Crom S (2002). Ammonia Pulses and Metabolic Oscillations Guide Yeast Colony Development.. Mol Biol Cell.

[40] Gori K, Mortensen HD, Arneborg N, Jespersen L (2007). Ammonia production and its possible role as a mediator of communication for *Debaryomyces hansenii* and other cheese-relevant yeast species.. J Dairy Sci.

[41] Bogonez E, Machado A Satrustegui J (1983). Ammonia accumulation in acetate-growing yeast.. Biochim Biophys Acta.

[42] Palková Z, Váchová L, Gásková D, Kucerová H (2009). Synchronous plasma membrane electrochemical potential oscillations during yeast colony development and aging.. Mol Membr Biol.

[43] Desai MJ, Armstrong DW (2003). Separation, identification, and characterization of microorganisms by capillary electrophoresis.. Microbiol Mol Biol Rev.

[44] Xiong G, Aras O, Shet A, Key NS, Arriaga EA (2007). Analysis of individual platelet-derived microparticles, comparing flow cytometry and capillary electrophoresis with laser-induced fluorescence detection.. Analyst.

[45] Řičicová M, Kučerová H, Váchová L, Palková Z (2007). Association of putative ammonium exporters Ato with detergent-resistant compartments of plasma membrane during yeast colony development: pH affects Ato1p localisation in patches.. Biochim Biophys Acta, Biomembr.

[46] Gietz RD, Schiesti RH, Willems AR, Woods RA (1995). Studies on the transformation of intact yeast cells by the LiAc/SS-DNA/PEG procedure.. Yeast.

[47] Wach A (1996). PCR-synthesis of marker cassettes with long flanking homology regions for gene disruptions in *S. cerevisiae*.. Yeast.

[48] Preston RA, Murphy RF, Jones EW (1989). Assay of vacuolar pH in yeast and identification of acidification-defective mutants.. Proc Natl Acad Sci U S A.

[49] Ali R, Brett CL, Mukherjee S, Rao R (2004). Inhibition of sodium/proton exchange by a Rab-GTPase-activating protein regulates endosomal traffic in yeast.. J Biol Chem.

[50] Wojdyr M (2010). Fityk: a general-purpose peak fitting program.. J Appl Crystallogr.

