# Supplementary figures and images for: In Vivo Determination of Organellar pH Using a Universal Wavelength-Based Confocal Microscopy Approach
Source: PLoS One. 2012 Mar 21;7(3):e33229. doi: 10.1371/journal.pone.0033229 (PMC3310042; doi:10.1371/journal.pone.0033229)

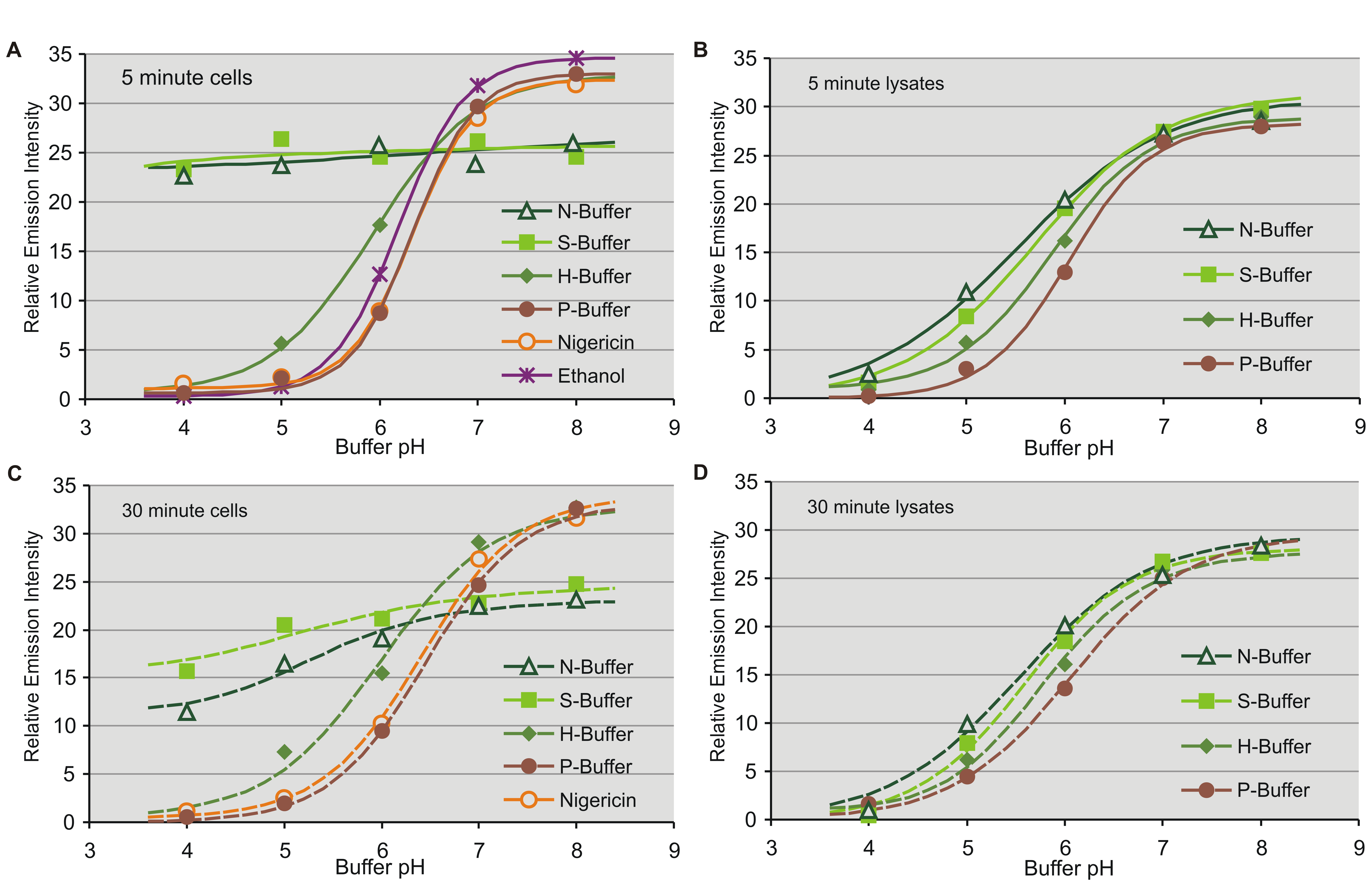

Supplement: Figure S1 — Spectrofluorometer intensity titrations. Emission intensity (unitless) versus pH titration curves from Ato1p-yEGFP1 fresh cells and cell lysate in calibration buffers of pH 4–8 with increasing permeating capacity (N, S, H and P-buffer). Titration curves of fresh cells (A) and cell lysate (B) after 5 min in suspension. Titration curves of fresh cells (C) and cell lysate (D) after 30 min in suspension. Cell lysis and treatment with high-dose ethanol (60%) before N-buffer suspension were included as invasive controls, while treatment with 10 µM nigericin before P-buffer suspension was a positive permeabilizing control. Examination of the titration curves implied a connection between the sigmoid slope and the degree of cell permeation. The titration curve achieved after sodium azide addition (transition from H to P-Buffer) was comparable to that of cell lysate, nigericin and ethanol controls; suggesting efficient transmembrane H+ equilibration. (TIF) [file pone.0033229.s001.tif]

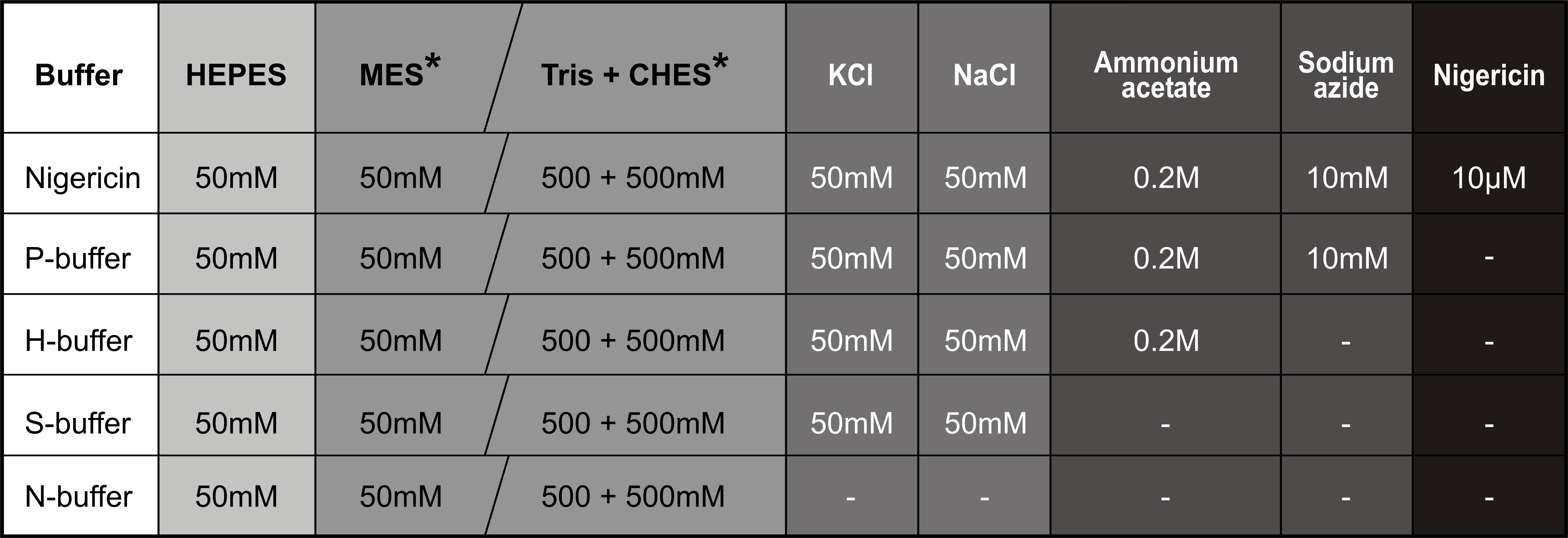

Supplement: Figure S2 — Titration buffer composition. Biological buffers HEPES and MES were employed in all titrations from pH 4 to 8. * For mCherry titrations from pH 6 to 11, MES was replaced with Tris and CHES to cover the higher alkali range. KCl and NaCl stabilized cells under long-term or permeant conditions. The combined interaction of ammonium acetate and sodium azide mediated the permeating capacity of the buffers. Nigericin was only added to the P-buffer solution in the positive permeabilizing controls. (TIF) [file pone.0033229.s002.tif]

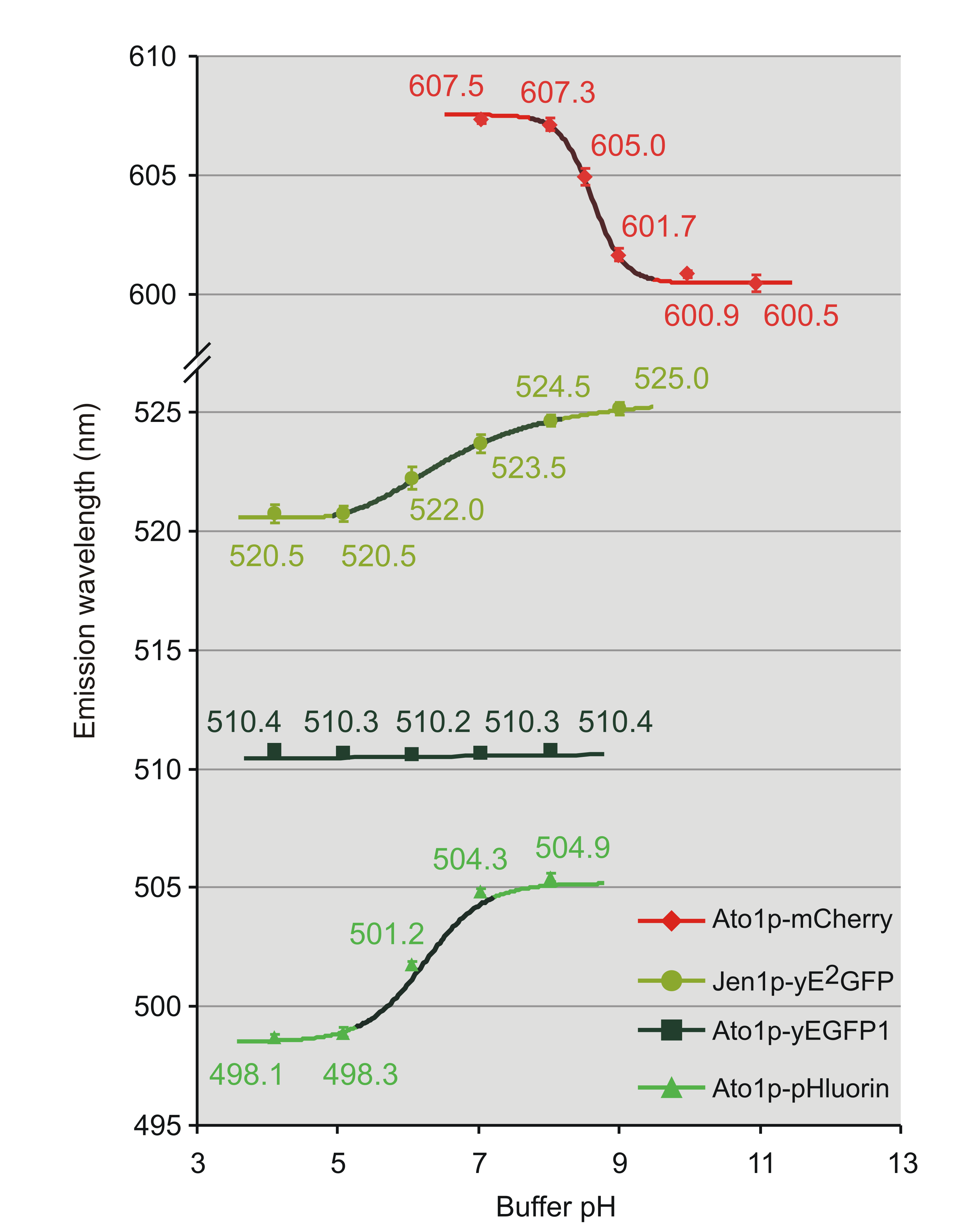

Supplement: Figure S3 — Confocal wavelength titrations. Emission wavelength (nm) versus pH titration curves from organellar ROIs within yeast cells suspended in P-buffer of pH 4–11. Strains expressing ratiometric pHluorin, yE2GFP and mCherry exhibited pH-dependent wavelength shifts indicative of ratiometry by emission at the given pH ranges. Representative values were averaged from individual subsets of 10 cells with 20 subcellular ROIs each. Error bars indicate the standard error. The darker areas within the sigmoidal titration curves represent their quantitation limits. (TIF) [file pone.0033229.s003.tif]

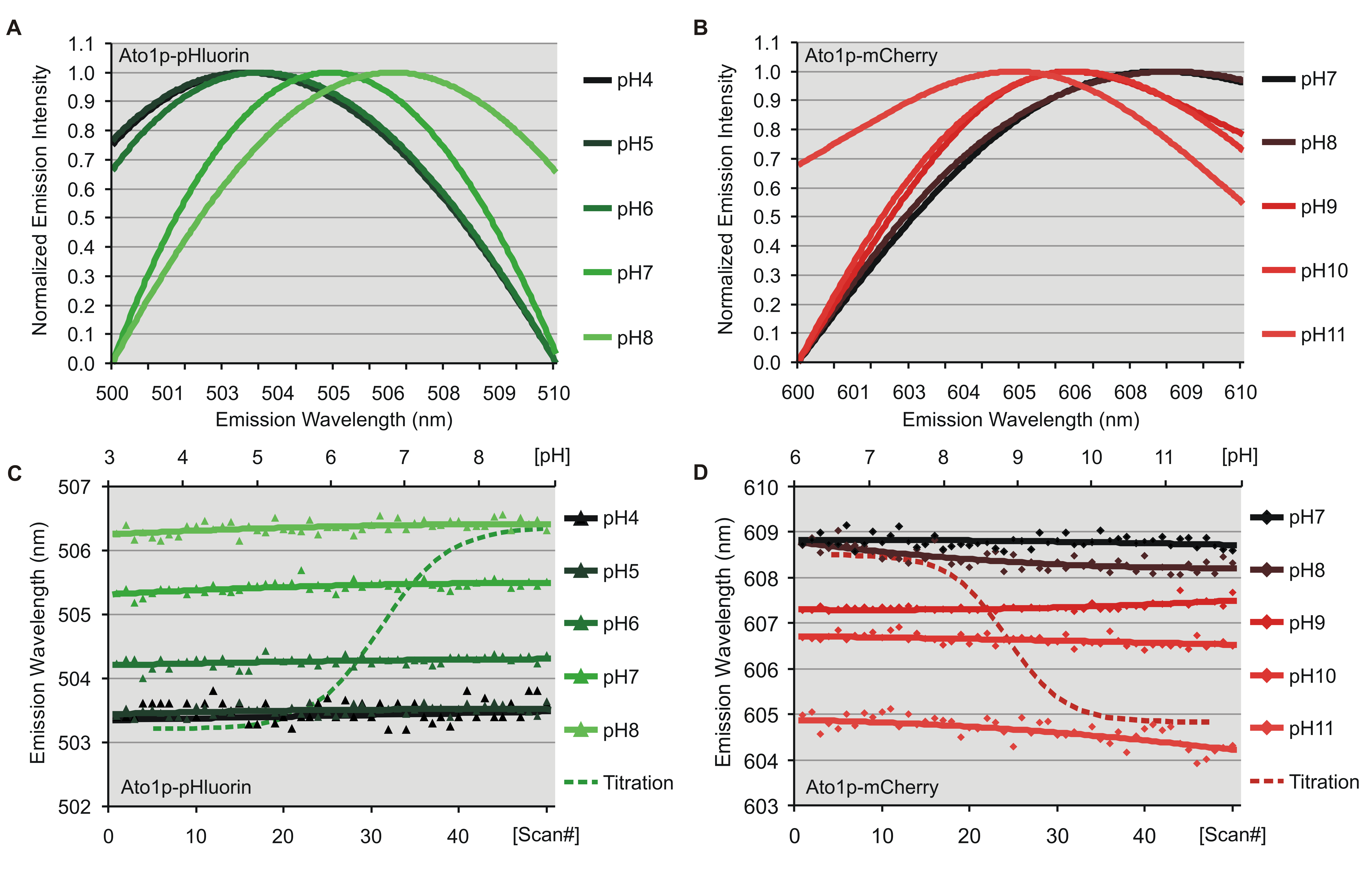

Supplement: Figure S4 — Spectrofluorometer wavelength stability tests. Emission spectra (nm) at different pH from Ato1p-pHluorin (A) and Ato1p-mCherry (B) fresh cells suspended in P-buffers of pH 4–11. Emission intensities were normalized to better show peak shifts. Wavelength stability curves (solid lines) from Ato1p-pHluorin (C) and Ato1p-mCherry (D) fresh cells illustrate the absence of emission spectral fluctuations throughout 50 successive spectrofluorometric scans under different pH conditions. Preliminary spectrofluorometer wavelength titrations (dashed lines) projected over the additional top x-axis (pH) are included for comparison. (TIF) [file pone.0033229.s004.tif]

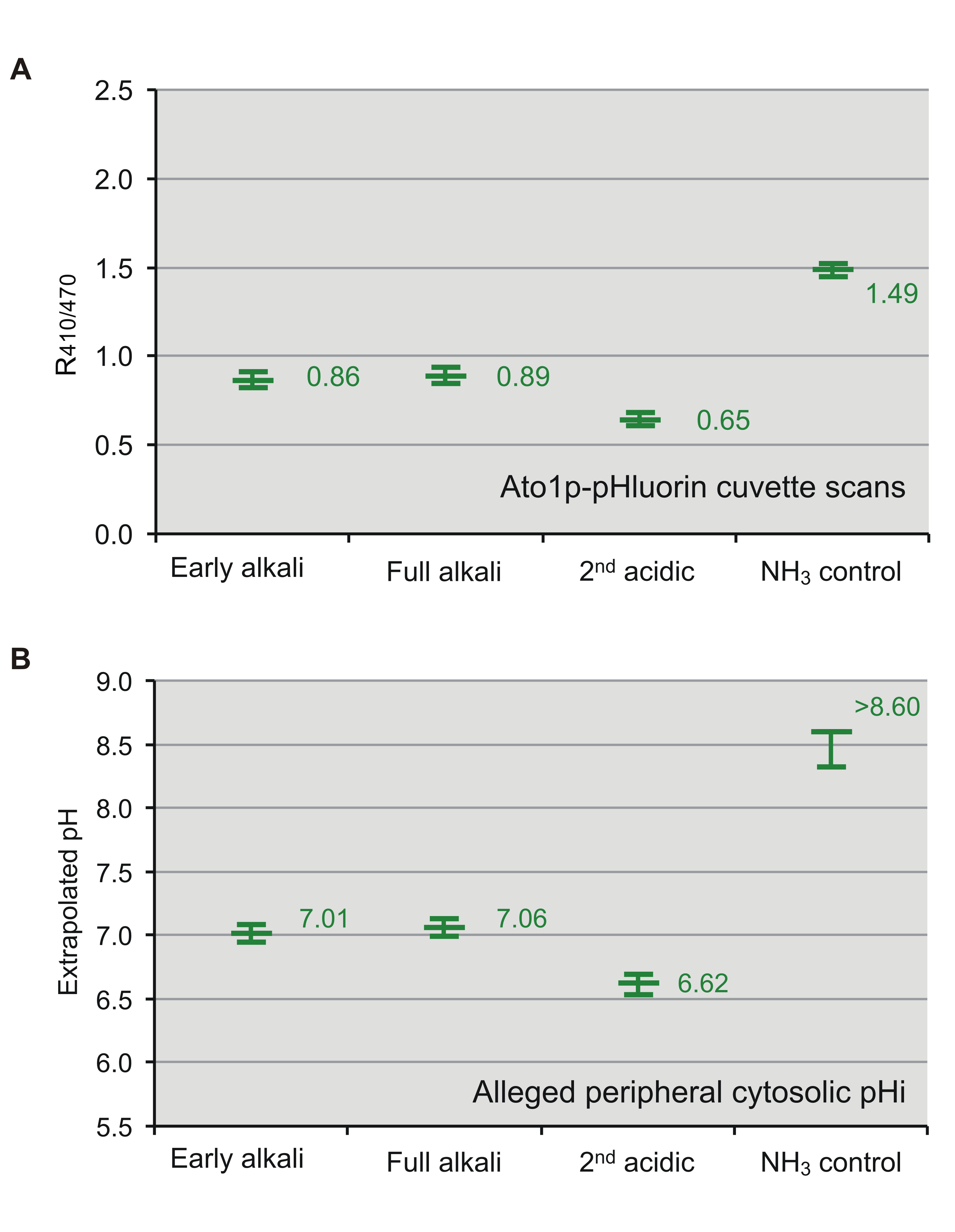

Supplement: Figure S5 — Spectrofluorometer dual ratio-based pH quantifications. (A) Dual ratios (R410/470) from peripheral ROIs within Ato1p-pHluorin bulk cell populations suspended in N-buffer of pH 6 under near-native conditions. Spectrofluorometric scans under excitation at 410 and 470 nm were performed during alkali and 2nd acidic phases. 2nd acidic cells treated with NH3 served as an alkalinization positive control. Each sample subset was constituted by a total of 22 sequential scans from 2 independent biological repeats. Error bars do not indicate variability within the colony but spectrofluorometric measurement standard error. In comparison to the CFM wavelength-based approach, spectrofluorometric dual ratio-based measurements were not sensitive enough to sufficiently detect physiological pHi changes. (B) Titration-based pH extrapolations from Ato1p-pHluorin (R410/470) data. The effective range of the extrapolations was strictly defined by the pKa and quantitation limits of the titrations. (TIF) [file pone.0033229.s005.tif]

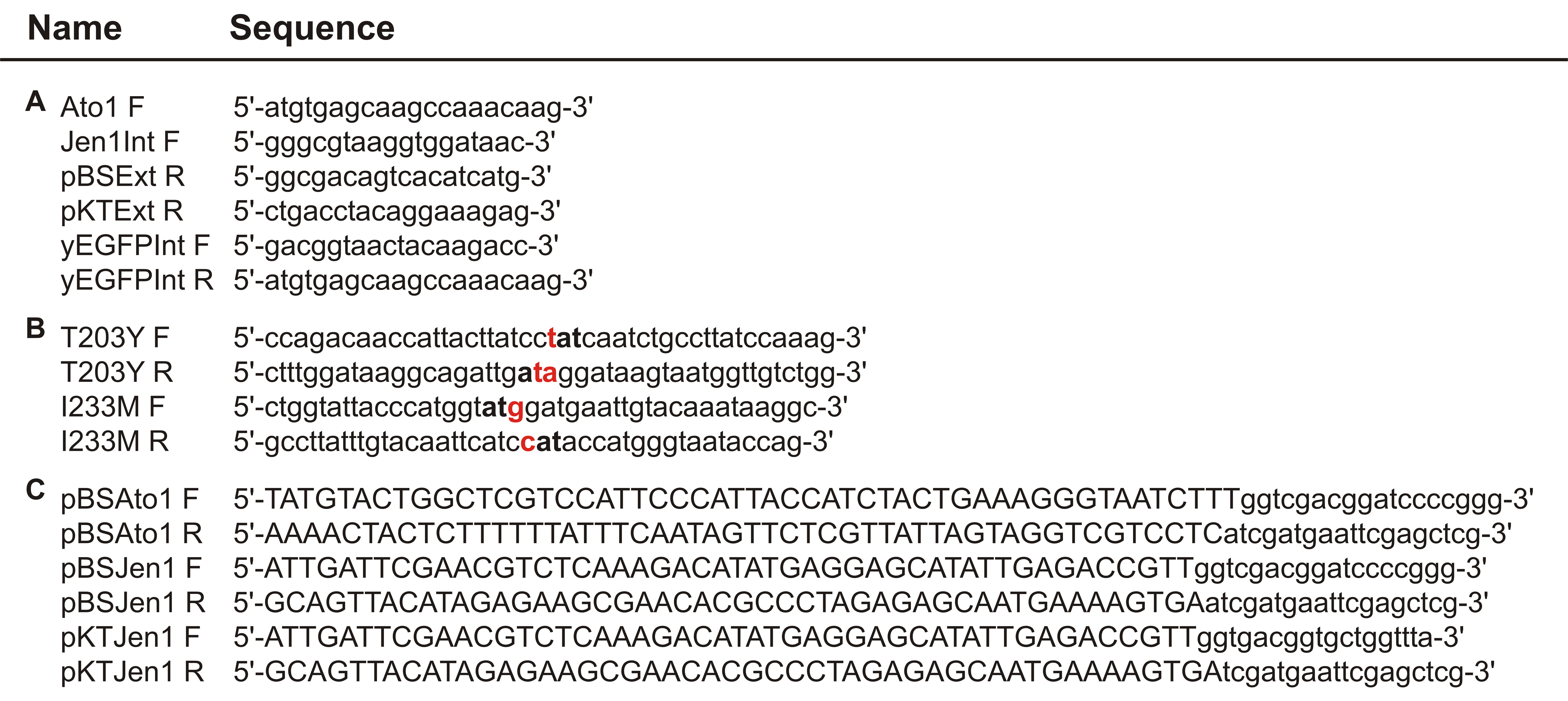

Supplement: Figure S6 — Primer list. Primer sequences are presented in 5′ to 3′ orientation. PCR primers (A) are shown in lower case. Mutagenic primers (B) contain 5′ and 3′ end sequences homologous to the target gene (lower case) and codon substitutions (bold case) involving 1–2 bp changes (red case). Cassette primers (C) contain 5′ end sequences homologous to the target gene (upper case) and 3′ end sequences homologous to the template plasmid (lower case). (TIF) [file pone.0033229.s006.tif]
