# Supplementary material for: In Vivo Determination of Organellar pH Using a Universal Wavelength-Based Confocal Microscopy Approach
Source: PLoS One. 2012 Mar 21;7(3):e33229. doi: 10.1371/journal.pone.0033229 (PMC3310042; doi:10.1371/journal.pone.0033229)
Supplement: File S1 — Supporting Information. (DOC) [file pone.0033229.s007.doc]

**SUPPORTING INFORMATION**

**In Vivo Determination of Organellar pH Using a Universal Wavelength-Based Confocal Microscopy Approach**

Albert Pineda Rodó, Libuše Váchová and Zdena Palková

**Supporting Information**

Buffer optimization based on ecliptic intensity titrations

Spectrofluorometric analyses of *S. cerevisiae* BY4742 expressing *ATO1* C-terminally tagged with ecliptic variants yEGFP1 or yEGFP3 were performed using different pH buffers to calculate intensity titration curves for the optimization of buffer composition. For the positive permeabilizing control, cells were treated with 10μM nigericin before P-buffer suspension. For the invasive controls, cells were either treated with high-dose ethanol (60%) before N-buffer suspension or lysed by glass bead disruption as previously described [1]. The ecliptic probes showed constant emission wavelengths at 508 and 512 nm respectively and pH-dependent intensity fluctuations which led to Boltzmann sigmoidal titration curves with apparent pKa values of 6.3 for yEGFP1 and 6.5 for yEGFP3. Fluorescence intensities increased with alkaline pH, with yEGFP1 reaching quantum yields 50-fold higher than yEGFP3. Ato1p-yEGFP1 cells in N-buffer retained constant intensities independently of external pH, resulting in horizontal titration curves. Only after prolonged periods in high acidity (pH<5) did the signal decrease (Figures S1, *A* and *C*). Ato1p-yEGFP1 fresh cells in P-buffer responded to pH in a sigmoidal fashion comparable to those exhibited by nigericin-permeated cells and invasive controls subjected to glass bead lysis or high-dose ethanol (Figures 1, *A* and S1). Titrations with intermediate buffers implied that efficient and time-stable (≥30 minutes) transmembrane H^+^ equilibration was achieved after sodium azide addition and further indicated that the sigmoid slope is connected to the degree of cell permeation (for detailed buffer composition, see Figure S2). Accordingly, titrations from lysate stayed sigmoidal regardless of buffers or treatment times (Figure S1).

Confocal laser scanning microscopy provided fluorescent spectra within separate subcellular regions of interest (ROIs) as well as strong evidence that invasive permeabilizing methods can seriously compromise the integrity of vacuoles and probably other organelles. Fluorescence intensities were collected from vacuoles (vacuolar ROIs) and the peripheral cytoplasm adjacent to the plasma membrane (peripheral ROIs). Likewise spectrofluorometer-based titrations, CFM intensity titration curves from Ato1p-yEGFP1 cells suspended in N-buffer remained horizontal within the pH and time range needed for our measurements; an indication of cells not responding to change in external pH. Titration curves from peripheral and vacuolar ROIs within cells in P-buffer (Figure 1, *B*-*C*) adopted a sigmoidal shape with matching pKa and differences in the extent of the pH-dependent intensity changes (the height of the sigmoid curve) mainly attributable to the intrinsic brightness of each compartment. Relative intensities acquired by CFM were purposely not normalized in order to show these differences and also pH-unrelated intensity artifacts: Confocal intensity titrations from cells under high ethanol doses followed sigmoidal curves with similar pKa. However, and unlike spectrofluorometric titrations (Figure 1, *A*), they were able to reveal the expected adverse effects of the invasive treatment: namely altered properties of ecliptic yEGFP1, as implied by the extended sigmoid height (Figure 1, *B*) and more importantly, the severe disruption of all subcellular structures (Figure 1, *D*).

Detailed strain construction and selection

For the preparation of BY-Ato1p-mCherry and BY-Jen1p-mCherry strains, two recombination cassettes were PCR-amplified using primers pBSAto1 F/R or pBSJen1 F/R and template plasmid pBS35 (Yeast Resource Center) [2] harboring mCherry, HphR and AmpR. The mCherry-HphR cassettes were inserted at the C-terminus of endogenous Ato1p or Jen1p [3]. Transformation of the *Saccharomyces cerevisiae* BY4742 cells was achieved by the lithium acetate method [4]. Correct integration was verified by sequencing with Ato1 F or Jen1Int F and pBSExt R. *S. cerevisiae* single colonies transformed with mCherry-HphR cassettes were selected at 28°C on GMA plates with 300µg/mL hygromycin-B.

For the mutagenesis of yeast codon-optimized yE^2^GFP, template plasmid pKT209 (EUROSCARF) harboring yEGFP1 (F64L/S65T/M233I) [5], CaURA3 and AmpR, was amplified with primers T203Y F/R and I233M F/R to introduce mutations T203Y and I233M in accordance with yeast codon usage. Plasmids were then transformed into *Escherichia coli* XL10 Gold competent cells (Stratagene, La Jolla, CA). Single colonies expressing AmpR were selected at 37ºC on LB plates (2.5% nutrient broth nº2 (Imuna Pharm, Sarisske Michalany, Slovakia), 2% agar) with 100μg/ml ampicillin. The successful mutation of yEGFP1 into yE^2^GFP was confirmed through sequence analysis using internal primers yEGFPInt F/R. For the construction of the BY-Jen1p-yE^2^GFP strain, yE^2^GFP-CaURA3 cassettes were amplified with primers pKTJen1 F/R and transformed into *S. cerevisiae* BY4742 cells. Single colonies harboring the yE^2^GFP-CaURA3 cassettes were selected at 28°C on minimal medium plates (2% glucose, 2% agar, 1 µl/ml Wickerham solution, 3.8M (NH_4_)^2^SO_4_, 0.74M KH_2_PO_4_, 0.2M MgSO_4_*7H_2_O, 38mM leucine, 27mM lysine and 32mM histidine) lacking uracil. Correct integration was verified by sequencing with Jen1Int F and pKTExt R. All primer sequences are listed in Figure S6.

Transmembrane topology predictions

Target proteins Ato1p and Jen1p were analyzed with six in silico topology prediction models aiming to find consensus predictions of its relative plasma membrane orientation. Available programs base their computations on the amino acid sequence and hydrophobicity profiles. PREDICTPROTEIN [6] provided information on putative transmembrane helices. In addition to transmembrane predictions, PSORT II, TMPRED and HMMTOP [7,8,9] also estimated N-terminus topology, while TMHMM and TOPPRED [10,11] predicted both N- and C-terminal orientation.

**References**

1. Váchová L, Kučerová H, Devaux F, Ulehlová M, Palková Z (2009) Metabolic diversiﬁcation of cells during the development of yeast colonies. Environ Microbiol 11: 494-504.
2. [Riffle M,](http://www.refdoc.fr/?traduire=en&FormRechercher=submit&FormRechercher_Txt_Recherche_name_attr=auteursNom: (RIFFLE)) [Malmström L](http://www.refdoc.fr/?traduire=en&FormRechercher=submit&FormRechercher_Txt_Recherche_name_attr=auteursNom: (MALMSTRÖM)), [Davis](http://www.refdoc.fr/?traduire=en&FormRechercher=submit&FormRechercher_Txt_Recherche_name_attr=auteursNom: (DAVIS)) TN (2005) The yeast resource center public data repository. Nucleic Acids Res 33: 378-382.
3. Wach A (1996) PCR-synthesis of marker cassettes with long flanking homology regions for gene disruptions in *S. cerevisiae*. Yeast 12: 259-265.
4. Gietz RD, Schiesti RH, Willems AR, Woods RA (1995) Studies on the transformation of intact yeast cells by the LiAc/SS-DNA/PEG procedure. Yeast 11: 355-360.
5. Sheff MA, Thorn KS (2004) Optimized cassettes for ﬂuorescent protein tagging in *Saccharomyces cerevisiae*. Yeast 21: 661-702.
6. Rost B, Liu J (2003) The PredictProtein server. Nucleic Acids Res. 31: 3300-3304.
7. Nakai K, Horton P (1999) PSORT: a program for detecting sorting signals in proteins and predicting their subcellular localization. Trends Biochem Sci 24: 34-35.
8. Hofmann K, Stoffel W (1993) TMbase - A database of membrane spanning proteins segments. Biol Chem Hoppe-Seyler 347: 166-166.
9. Tusnády GE, Simon I (2001) The HMMTOP transmembrane topology prediction server. Bioinformatics 17: 849-850.
10. Sonnhammer ELL, Eddy SR, Durbin R (1997) Pfam: a comprehensive database of protein domain families based on seed alignments. Proteins: Struct Funct Bioinf 28: 405-420.
11. Claros MG, Von Heijne G (1994) TopPred II: an improved software for membrane protein structure predictions. Comput Appl Biosci 10: 685-685.
